# Potential Involvement of Oxidative Stress in Ligamentum Flavum Hypertrophy

**DOI:** 10.3390/jcm12030808

**Published:** 2023-01-19

**Authors:** Kei Ito, Hideki Kise, Satoshi Suzuki, Sota Nagai, Kurenai Hachiya, Hiroki Takeda, Soya Kawabata, Daiki Ikeda, Keiyo Takubo, Shinjiro Kaneko, Nobuyuki Fujita

**Affiliations:** 1 Department of Orthopaedic Surgery, School of Medicine, Fujita Health University, Toyoake 470-1192, Japan; 2 Department of Orthopaedic Surgery, Keio University School of Medicine, Tokyo 108-8345, Japan; 3 Department of Spine and Spinal Cord Surgery, School of Medicine, Fujita Health University, Toyoake 470-1192, Japan; 4 Department of Stem Cell Biology, Research Institute, National Center for Global Health and Medicine, Tokyo 162-8655, Japan

**Keywords:** lumbar spinal canal stenosis, ligamentum flavum hypertrophy, oxidative stress, reactive oxygen species, N-acetylcysteine

## Abstract

Oxidative stress (OS) results in many disorders, of which degenerative musculoskeletal conditions are no exception. However, the interaction between OS and ligamentum flavum (LF) hypertrophy in lumbar spinal canal stenosis is not clearly understood. The first research question was whether OS was involved in LF hypertrophy, and the second was whether the antioxidant N-acetylcysteine (NAC) was effective on LF hypertrophy. In total, 47 LF samples were collected from patients with lumbar spinal disorders. The cross-sectional area of LF was measured on axial magnetic resonance imaging. Immunohistochemistry of 8-OHdG and TNF-α were conducted on human LF samples. A positive association was found between 8-OHdG or TNF-α expression and cross-sectional area of LF. Flow cytometry analysis showed that H_2_O_2_, buthionine sulfoximine, and TNF-α treatment significantly increased intracellular reactive oxygen species in primary LF cells. NAC inhibited the induction of LF hypertrophy markers by OS or TNF in a real-time reverse transcriptase polymerase chain reaction and enzyme-linked immunosorbent assay. Western blotting analysis indicated that p38, Erk, and p65 phosphorylation were involved in intracellular OS signaling in LF cells. In conclusion, our results indicated that OS could be a therapeutic target for LF hypertrophy. Although this study included no in vivo studies to examine the longitudinal efficacy of NAC on LF hypertrophy, NAC may have potential as a therapeutic agent against lumbar spinal canal stenosis.

## 1. Introduction

Lumbar spinal canal stenosis (LSS) is a highly prevalent degenerative musculoskeletal disease, which leads to a limitation of functional activity for patients with pain in the low back and lower extremities. With the increasing proportion of the geriatric population, LSS has started to show an upward trend in incidence along with financial burden and social participation restrictions [1,2,3,4]. Presently, LSS treatment is limited to conservative treatment to control low back and lower extremity pain or surgical treatment for enlargement of the lumbar spinal canal and fixation of spinal instability [5,6,7,8]. The pathological changes associated with LSS are commonly due to ligamentum flavum (LF) hypertrophy, degenerative changes in the intervertebral disc (IVD), and facet joint osteophyte formation. These changes can potentially cause chronic cauda equina and nerve root impingement. The changes associated with LF hypertrophy are elastin fiber proportion reduction and an increase in collagen fibers, particularly type I and type III collagen, which increase proportionately with LF hypertrophy [9,10,11]. Meanwhile, a recent histological study showed that the total cell number was significantly higher in the hypertrophied LFs than in the non-hypertrophied LFs [12]. Therefore, the pathology of LF hypertrophy includes both hyperplasia and an increase in extracellular matrix.

Regarding the LF hypertrophy mechanisms, mechanical loading, macrophage infiltration, and chronic inflammation were documented to be involved [13,14,15,16,17,18]. In LF hypertrophy, there is a particular increase in proinflammatory cytokines like tumor necrosis factor (TNF)-α and interleukin (IL) 1-β, which can disturb the normal collagen and elastin fiber proportion [15,16]. However, the molecular aspect is not clearly understood and explored even after a good research volume on LF hypertrophy [13,14,15,16,17,18].

As per the free-radical theory, which defines the aging changes, oxidative stress (OS) caused by reactive oxygen species (ROS) causes a functional decline in the aging population [19]. OS causes many disorders, among which are degenerative musculoskeletal conditions like osteoarthritis and osteoporosis [20,21,22]. Several studies have reported that IVD degeneration, one of the etiological factors of LSS, is also related to OS [23,24,25]. We also previously reported that OS was involved in IVD degeneration progression and that N-acetylcysteine (NAC), one of the antioxidants, may inhibit IVD degeneration progression [26]. NAC is a vital drug for treating acetaminophen overdose and a widely used dietary supplement. Meanwhile, a limited number of papers have analyzed the interaction between OS and LF hypertrophy [27,28]. Thus, in this study, the first research question was whether OS was involved in LF hypertrophy, and the second was whether NAC is effective in LF hypertrophy.

## 2. Materials and Methods

### 2.1. Human LF Samples

This study was performed with 47 LF tissue samples obtained from participants who were surgically treated for lumbar spinal disorders after obtaining informed consent to utilize them for our research. The ethics committee of our institution approved this study. A portion of the samples have been used in previous studies [29,30]. Table 1 presents the participants’ baseline characteristics.

#### Magnetic Resonance Imaging

**Figure 1:**
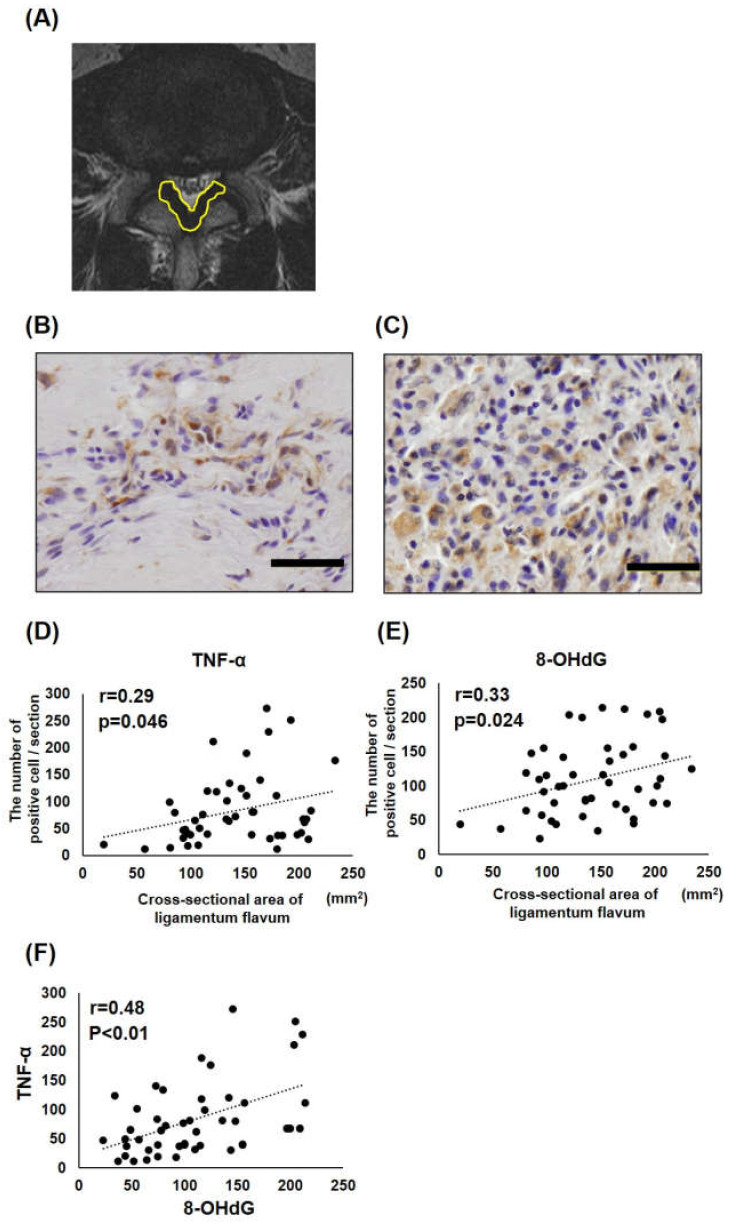
Correlation between 8-OHdG or TNF-α expression and ligamentum flavum (LF) hypertrophy. (**A**) Measurement of the cross-sectional area (CSA) of LF under axial MRI. (**B**,**C**) Representative immunohistochemistry data for TNF-α (**A**) and 8-OHdG (**B**) in human LF samples. Scale bars, 50 μm. (**D**,**E**) Correlation between the positive cell number of TNF-α (**C**) or 8-OHdG (**D**) and the LF cross-sectional area on axial lumbar magnetic resonance imaging. (**F**) Correlation of the positive cell number of TNF-α and 8-OHdG.

According to previous studies [29], preoperative lumbar magnetic resonance imaging (MRI) was used to measure the LF’s cross-sectional area (CSA) at the level from which LF samples were obtained during surgery (Figure 1a).

### 2.2. Immunostaining

Antibodies for TNF-α (diluted 200-fold; Novus Biologicals, Centennial, CO, USA) and 8-hydroxy-2′-deoxyguanosine (8-OHdG) (diluted 200-fold; Japan Institute for the control of Aging, Shizuoka, Japan) were used for immunohistochemistry. A peroxidase-labeled anti-rabbit antibody (Histofine Simplestain Max PO, Nichirei, Tokyo, Japan) was followed as the secondary antibody.

### 2.3. Human LF Cells

Human LF cells were isolated, as reported earlier [30]. The cells were put into 6-well plates before treatment with reagents at a density of 1 × 10^5^ cells/well and 10 cm dishes at a density of 5 × 10^5^ cells/dish for real-time reverse transcriptase polymerase chain reaction (RT-PCR) and Western blotting (WB), respectively. In the in vitro experiments, the original samples were not used, and LF cells were extracted from the surgical LF samples of a 68-year-old woman diagnosed with lumbar disc herniation (LDH) at L 4/5 level. The isolated LF cells were used in five passages for in vitro analyses.

### 2.4. Measurement of Intracellular ROS

The dissociated cells were loaded with Mitotracker Orange CMH2TM ROS (Thermo Fisher Scientific, Waltham, MA, USA) and incubated on a shaker at 37 °C for 30 min. FACSAria was used (Becton, Dickinson, and Company, Franklin Lakes, NJ, USA) for intracellular ROS measurement.

### 2.5. Treatment

Primary LF cells were treated with H_2_O_2_ (100 μM; Wako, Tokyo, Japan), buthionine sulfoximine (BSO) (1 mM; Sigma-Aldrich, St. Louis, MO, USA), which is a glutathione synthesis suppressor that initiates OS, recombinant human TNF-α (50 ng/mL; Thermo Fisher Scientific, Waltham, MA, USA) and NAC (100 uM; Sigma-Aldrich) for 24 h. The LF cells were cultured with TNF α (50 ng/mL) and BSO (1 mM) for a duration of 30 min in association with NAC pretreatment (100 uM, 10 min) to assess the phosphorylation capacity of intracellular signaling. These experiments were conducted independently in triplicate.

### 2.6. Real-Time RT-PCR

Real-time RT-PCR was conducted according to the previous report [30]. Type I and type III collagen, TNF-α, and β-actin primers were used and the results were quantified using the ddCt.

### 2.7. WB Analysis

The following antibodies were utilized: nuclear factor (NF)-κB p65, phosphorylated p65, extracellular signal-regulated kinases (Erk), phosphorylated Erk, c Jun amino-terminal kinase (JNK), phosphorylated JNK, p38, and phosphorylated p38. All these antibodies were bought from Cell Signaling Technology (Danvers, MA, USA), which were diluted 1000-fold.

### 2.8. Enzyme-Linked Immunosorbent Assay (ELISA)

Human Pro-Collagen I alpha 1 ELISA Kit (Bio-Techne, Minneapolis, MN, USA) was used for quantitative type I collagen measurement in cell culture supernatants.

### 2.9. Statistical Analysis

The mean ± standard deviation was the central tendency, and dispersion was preferred to specify the data. A parametric independent *t*-test, nonparametric Mann–Whitney U test, parametric analysis of variance, or nonparametric Kruskal–Wallis were used for the corresponding data type. A significance level of *p* < 0.05 was used for the study. Pearson’s correlation coefficient was used to analyze the relationship between the CSA of LF on MRI and the total positive cell number of TNF-α or 8-OHdG in 100 fields.

## 3. Results

### 3.1. Expression of TNF-α and 8-OHdG in LF Tissues

To evaluate the chronic inflammation and OS level in hypertrophied LF, TNF-α, and 8-OHdG immunohistochemistry was conducted in LF samples, respectively. Subsequently, the positive TNF-α or 8-OHdG cells in 100 fields of each section were counted. Figure 1b,c shows high protein expression of TNF-α and 8-OHdG in hypertrophied LF, respectively. Because even patients who were diagnosed with LDH may have LF hypertrophy clinically, we did not compare LF samples of LDH and LSS but rather we put these samples together and analyzed the correlation between their values in the present study. The correlation between the positive cell numbers of TNF-α and 8-OHdG with LF CSA out of the 47 samples was examined (Figure 1d,e). Both TNF-α and 8-OHdG showed a significant but weak correlation with the CSA of LF (TNF-α; r = 0.29, *p* = 0.046; 8-OHdG; r = 0.33, *p* = 0.024). Furthermore, there was a moderate correlation between TNF-α and 8-OHdG positive cell numbers (r = 0.48, *p* < 0.01) (Figure 1f).

### 3.2. Intracellular ROS Level in LF Cells

**Figure 2:**
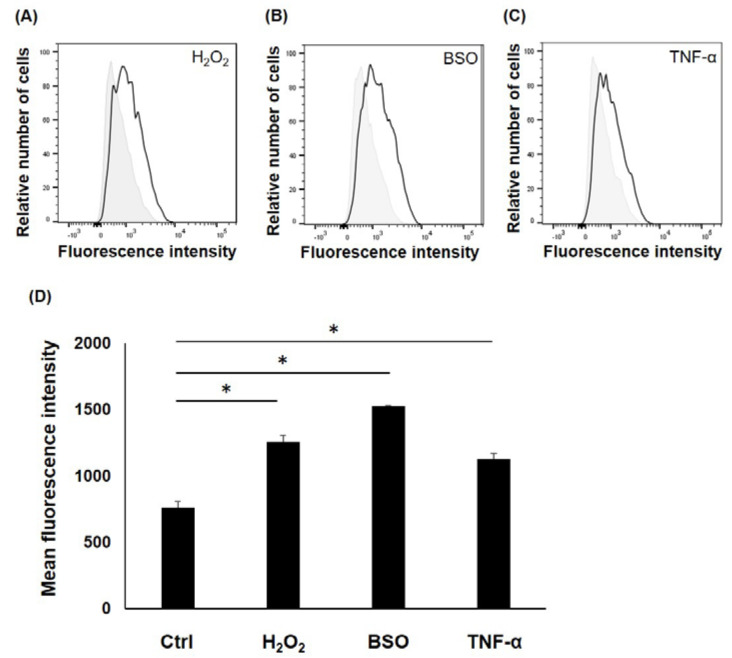
Flow cytometry analysis for the intracellular level of reactive oxygen species (ROS) in ligamentum flavum (LF) cells. (**A**–**C**) The horizontal axis represents the fluorescence intensity of the ROS probe per cell, and the vertical axis represents the relative cell number at each fluorescence intensity. Histograms filled in gray represent fluorescence data for untreated control cells, while solid black lines represent H_2_O_2_-treated (**A**), buthionine sulfoximine (BSO)-treated (**B**), and TNF-α-treated cells (**C**). (**D**) Treatment of H_2_O_2_, BSO, and TNF-α significantly increased intracellular ROS levels in the cells. * <0.05.

The intracellular ROS level of LF cells cultured with H_2_O_2_, BSO, and TNF-α were analyzed using flow cytometry. Figure 2a–c shows representative data for the analysis of intracellular ROS. Figure 2d shows that H_2_O_2_, BSO, and TNF-α treatment significantly increased intracellular ROS levels in LF cells compared with the control.

### 3.3. Interaction between OS and Expression of LF Hypertrophy Markers in LF Cells

**Figure 3:**
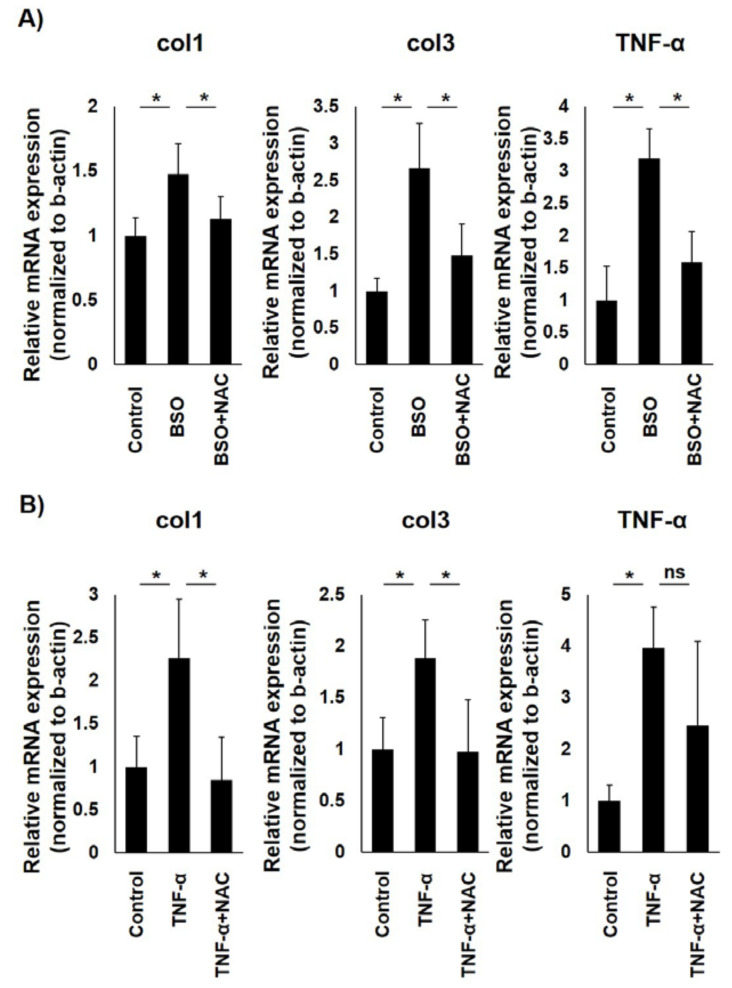
Real-time RT-PCR analysis to assess oxidative stress impact on ligamentum flavum (LF) cells. (**A**) Buthionine sulfoximine (BSO)-mediated induction of type I and type III collagen and TNF-α mRNA expression was significantly neutralized by N-acetylcysteine (NAC) treatment in LF cells. (**B**) NAC treatment significantly reduced TNF-α-mediated induction of type I and type III collagen mRNA expression in LF cells. These experiments were conducted independently in triplicate. Data are presented as the mean ± standard deviation. * <0.05.

To find the influence of OS on LF hypertrophy in vitro, type I and type III collagen and TNF-α mRNA expression were probed, which were greatly induced in LF hypertrophy, following BSO treatment of primary LF cells. For the suppressing experiment, NAC was added to BSO-treated LF cells. Figure 3a shows that BSO-mediated mRNA expression induction of type I and type III collagen and TNF-α was significantly reduced by NAC treatment in LF cells. TNF-α was administered to the LF cells with or without NAC, considering that TNF-α treatment induced the LF cells’ intracellular ROS level. Expectedly, TNF-α-mediated mRNA expression induction of type I and type III collagen was significantly reduced by NAC treatment in LF cells (Figure 3b). TNF-α-mediated induction of TNF-α mRNA expression was also reduced, albeit not significantly, by NAC treatment.

### 3.4. Intracellular Signaling of OS in LF Cells

**Figure 4:**
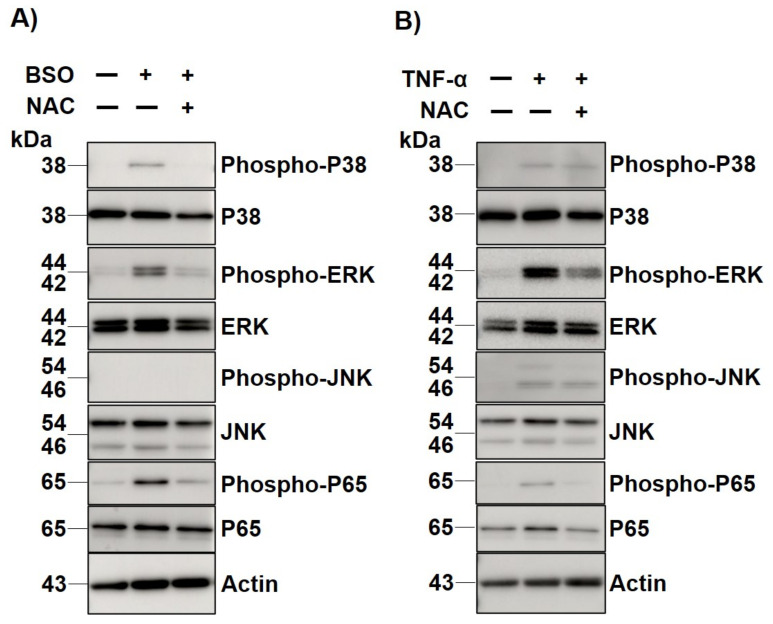
Western blot analysis for BSO- or TNF-α-mediated MAPKs and NF-κb p65 phosphorylation. (**A**) MAPKs phosphorylation, including p38, Erk 1/2, and p65, was enhanced after buthionine sulfoximine (BSO) treatment, but was suppressed by additional N-acetylcysteine (NAC) treatment. (**B**) TNF-α mediated Erk 1/2 and p65 phosphorylation was neutralized by NAC treatment, but not p38 and JNK.

The p38, Erk, JNK, and p65 phosphorylation status was evaluated in human LF cells following 30 min of BSO treatment with and without 10 min of NAC pretreatment to identify the intracellular signaling associated with the OS of LF hypertrophy. WB analysis clarified that p38, Erk 1/2, and p65 phosphorylation were enhanced by BSO treatment (Figure 4a). Apart from this, it was also observed that the phosphorylation of these molecules was suppressed when NAC was added (Figure 4a). Next, this study evaluated the phosphorylation status of TNF-α treated LF cells with/without NAC to determine whether TNF α mediated intracellular signaling is modulated by OS. WB analysis revealed that TNF-α mediated phosphorylation of Erk 1/2 and p65 was clearly neutralized by NAC treatment (Figure 4b).

### 3.5. Interaction between the OS and the Release of Type I Collagen in LF Cells

**Figure 5:**
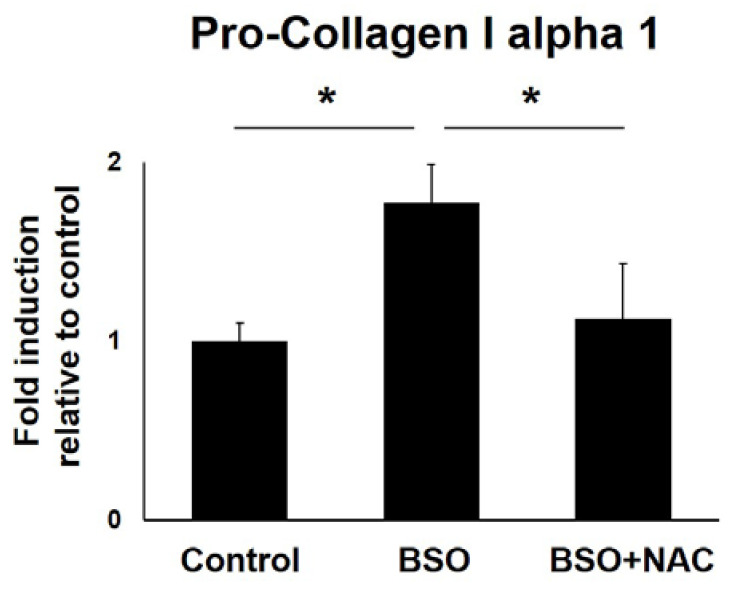
Enzyme-linked immunosorbent assay for quantitative pro-collagen I alpha 1 measurement in LF cell culture supernatants. Buthionine sulfoximine (BSO)-mediated induction of pro-collagen I alpha 1 was significantly reduced with N-acetylcysteine (NAC) treatment. These experiments were conducted independently in triplicate. The data are presented as the mean ± standard deviation. * < 0.05.

Finally, this study quantitatively measured the protein level of pro-collagen I alpha 1 in LF cell culture supernatants without or with BSO, or with BSO and NAC, to identify OS involvement in type I collagen release in human LF cells. ELISA showed that the BSO mediated induction of pro-collagen I alpha 1 was significantly reduced with NAC treatment (Figure 5).

## 4. Discussion

In this study, a positive association was established between 8-OHdG or TNF-α expression and LF hypertrophy. The functional outcome illustrated that treatment using NAC neutralized the OS-mediated induction of the mRNA expression of LF hypertrophy markers. WB analysis proved that p38, Erk, and p65 were the key components in OS in LF cells as a predominant intracellular signal agent. NAC treatment suppressed all the signaling phosphorylation. Additionally, NAC partially neutralized the TNF-α-mediated induction of the mRNA expression of LF hypertrophy markers and phosphorylation of intracellular signaling in LF cells. Finally, NAC reduced the OS mediated release of type I collagen in LF cells.

Our analysis showed a weak but significant positive correlation, between OS and LF hypertrophy, indicating that OS was potentially involved in LF hypertrophy. In this analysis, there were some concerns about reproducibility. Firstly, the LF CSA on MRI was measured at the most severely stenosed level from which the LF sample was taken, but it may not be completely consistent with the degree of LF hypertrophy because the LF CSA is expected to depend on other factors, such as the IVD level, the subject’s physique and gender, and pathological condition. Previous studies have used LF thickness on axial MRI as an LF parameter for hypertrophy, which has a similar problem [31]. In the currently available image modality, no parameter may be found that can completely represent LF hypertrophy. Under these situations, LF CSA on MRI may be the most common LF hypertrophy parameter, as used in past studies by our group and others [29,30,32]. Second, it should be noted that the 8-OHdG protein expression level in LF was measured semi-quantitatively from the immunostaining results, and thus this data is not a completely accurate representation of the OS amount. A method of quantitatively and reproducibly evaluating OS in the proteins directly extracted from LF tissues has not yet been established. Therefore, in the future, these results will have to be verified by further increasing the number of cases after matching the IVD level, gender, physique, and pathological condition.

Considering that there was a significant positive correlation between the expression levels of 8-OHdG and TNF-α in LF tissues and that TNF-α, as well as H_2_O_2_ and BSO, increased the intracellular LF cells’ ROS level in flow cytometry analysis, TNF-α can induce OS in LF cells. In real-time RT-PCR and WB analysis, the antioxidant NAC partially neutralized the influence caused by TNF-α, supporting these hypotheses. Conversely, it has been reported that OS induces chronic inflammation in other cells [26,33], suggesting that OS and chronic inflammation may also form a positive feedback loop. Furthermore, inflammatory cytokines, such as TNF-α and IL 1β, have also interacted with OS in another spine tissue, the IVD [26], suggesting that chronic inflammation and OS may be closely related throughout the LSS based on IVD degeneration and LF hypertrophy.

**Figure 6:**
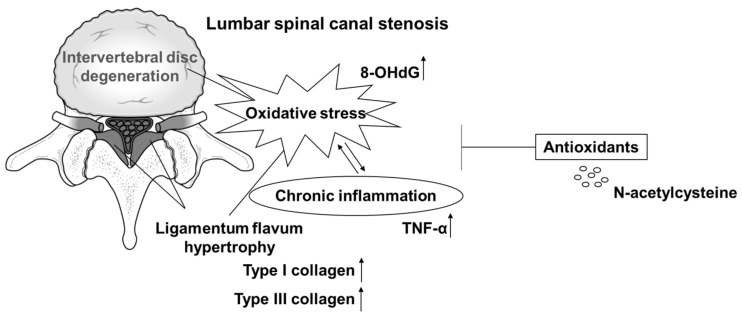
Schematic model showing the potential of N-acetylcysteine (NAC) as a preventive or therapeutic agent for lumbar spinal canal stenosis.

NAC has been used to treat a variety of diseases and is widely used as an antioxidant supplement [34,35]. Our results, although an in vitro experiment, also suggested that NAC was effective in suppressing LF hypertrophy. Recently, Hsu et al. also reported that NAC administration regressed the fibrogenic and proinflammatory OS effects in hypertrophic LF cells [28]. In their in vitro experiments, the concentration of NAC was 10 mM, while in our study, we used NAC at 100 μM. Given the similar results in the two studies, our results are of clinical significance, showing that even a low concentration of NAC is sufficiently effective. In addition, using animal models, OS was previously reported to induce IVD degeneration, and NAC has an inhibitory effect [26]. Combining present and past results, it is believed that NAC may have potential as a preventive or therapeutic agent against LSS, which is mainly caused by IVD degeneration and LF hypertrophy (Figure 6). NAC is a drug that is both medically and economically easy to prescribe for patients because it has few side effects and is inexpensive. However, because we have a number of concerns that need to be addressed, such as the dose, duration, and timing of oral administration, further preclinical and clinical evaluations are warranted.

The present study has several limitations. First, the number of human LF samples was limited. Second, as stated above, a technical bias was identified in the LF CSA measurements on MRI and the counting of 8-OHdG- or TNF-α-positive cells in the immunostained section. Lastly, no in vivo studies have been conducted to determine whether OS is involved in LF hypertrophy or whether NAC can suppress LF hypertrophy.

## 5. Conclusions

Our human sample analysis and in vitro analysis indicated that OS could be a therapeutic target for LF hypertrophy. Although the present study included no in vivo studies to examine the longitudinal efficacy of NAC on LF hypertrophy, NAC may have potential as a preventive or therapeutic agent against LSS.

## Figures and Tables

**Table 1:** Baseline characteristics of human surgical samples.

Samples		*n* = 47
Patients		*n* = 39
Gender	Male	18
	Female	21
Age (years)	≥80	3
	60–80	20
	40–60	10
	<40	6
Level	L2/3	2
	L3/4	9
	L4/5	24
	L5/S	12
Diagnosis	LSS	32
	LDH	15

LSS, lumbar spinal canal stenosis; LDH, lumbar disc herniation.

## Data Availability

The datasets generated and/or analyzed during the current study are not publicly available due to limitations of ethical approval involving the patient data and anonymity but are available from the corresponding author on reasonable request.
